# Tumor in the Neck—Admission of Air into Vein—Death Forty Hours after Operation

**Published:** 1864-04

**Authors:** J. F. Miner


ART. IV.—*Tumor in the Neck—Admission of Air into Vein—Death forty hours after Operation.* By J. F. MINER, M. D.

Joseph Fisher, of Lancaster, Erie County, N. Y., presented for removal a large tumor, situated upon the left side of the neck, extending from about midway of the ear above, to under the clavicle below; posteriorly, to near the spinal processes of the cervical vertebræ, while anteriorly it reached to the trachea, overlaping it in the middle portion. It was movable and lobulated, appearing as if encysted. There was not great pain. It was of two years' standing, having attained this size in that short period. The general health had suffered from it, or other causes not discovered, and the patient presented a sallow, anæmic condition, and was unable to continue labor as a farmer. He was admitted to a private ward in the Buffalo General Hospital March 24th, and assisted by Drs. Lothrop and Brown and students of medicine, operation was made while fully under the influence of sulphuric ether. While attempting to enucleate that portion which extended down somewhat under the clavicle, the internal jugular vein was torn, or partially cut, and air immediately drawn into the vein by the respiratory effort. It appeared almost as much inhaled as if drawn into the trachea; it was as distinct and unmistakable. The vessel was immediately seized and successfully ligated without great loss of blood, not however until two or three distinct inhalations of air into the vein had created the expectation that respiration would perhaps cease almost immediately, as is sometimes the case after such admission of air. This was not the case, and with the exception of a constant cough, no immediate effects were produced. The operation was successfully completed, and the patient placed comfortably in bed, re-action being soon fully established. During most of the next day the patient appeared quite comfortable, conversed freeiy and naturally, and assured his friends that he was not in pain and felt well. Pulse 120 to 130 per minute; respiration hurried; bronchial rale over left lung, the right having natural respiratory murmer; cough continued severe. Towards night the symptoms increased in severity, and there was some difficulty of articulation and deglutition; respiration more hurried, and pulse more frequent, feeble, and irregular; death took place forty hours after the operation.

That air was admitted into the internal jugular vein there can be no doubt; that this admission of air was the cause of death, or even the cause of the cough, bronchial irritation, etc., etc., is certainly matter of some uncertainty. *Post mortem* examination could not be obtained, and had it been made, though it would have enabled us to give a more complete history of the case, it probably would have thrown very little if any light upon the immediate causes of death. The exposure and disturbance of the important nerves in this region, may have operated to embarrass respiration and paralyze the muscles of deglutition as directly and fatally as admission of air to the large veins, though this latter is a more fashionable way of explaining the termination of such a case. The facts that no immediate ill effects were produced by the admission of air, and that partial paralysis supervened, lead to the inference that possibly nervous connections were injured or destroyed, and that the unfavorable termination was due to this, rather than to the effects of admission of air to the veins. Possibly both these causes operated to produce this result.

"The veins through which air is usually spontaneously admitted in surgical operations, are those about the neck, particularly the external and internal jugular, and their immediate branches. It may also enter by the veins of the face, the axilla, and the chest. In one of Dr. Mott's cases it passed in through the facial vein during the removal of an enlarged parotid gland. Dr. Warren had an instance in which it was introduced by the subcapular vein; Clemot saw it enter by the veins of the chest; and Delpeck had a case where it gained admission by the axillary vein." The reason why the veins in these localities are more subject to this accident is, that they are under the influence of a suction action, during inspiration, owing to a tendency to the formation of a vacuum within the chest during the expansion of the lungs. This action is mostly confined to the veins at the root of the neck, and it is here that air is most liable to enter spontaneously, though it may pass in through the veins of other parts, and is equally injurious when admitted at greater distances from the heart and lungs. This accident has happened to many surgeons, and in some instances been attended by immediately fatal results. The immediate cause of death in these cases has been variously explained; it is remarkable what variety of opinions there is upon the subject among medical writers.

There was in this case none of the common effects of such accident, and it is by no means certain that the admission of air influenced the termination of the case.

The disturbance of the important nerves may have been the cause of the embarrassed respiration, and this together with the shock of operation, may perhaps partially account for the symptoms and termination.

The report of an unsuccessful operation is really of much more value—

is more instructive and suggestive than of many wholly successful cases. They are more valuable again because they are much more rare. Surgery which is successful, is reported with pride and pleasure, while the failures are concealed and suppressed. On this account we have reported the above, giving all an opportunity to judge for themselves as to the causes of failure or the conditions necessary to success.

